# Antibacterial Activity and Mechanism of Three Root Exudates from Mulberry Seedlings against *Ralstonia pseudosolanacearum*

**DOI:** 10.3390/plants13040482

**Published:** 2024-02-08

**Authors:** Ping Li, Siyi Wang, Mengyuan Liu, Xue Dai, Huicong Shi, Weihong Zhou, Sheng Sheng, Fuan Wu

**Affiliations:** 1 Jiangsu Key Laboratory of Sericultural Biology and Biotechnology, School of Biotechnology, Jiangsu University of Science and Technology, Zhenjiang 212100, China; lee_ping2020@163.com (P.L.); 221211802114@stu.just.edu.cn (S.W.); 211111802105@stu.just.edu.cn (M.L.); 231211801103@stu.just.edu.cn (X.D.); shihcc08@163.com (H.S.); zhouweihong2020@just.edu.cn (W.Z.); parasitoids@163.com (S.S.); 2 Key Laboratory of Silkworm and Mulberry Genetic Improvement, Ministry of Agriculture and Rural Affairs, The Sericultural Research Institute, Chinese Academy of Agricultural Sciences, Zhenjiang 212100, China

**Keywords:** bacterial wilt, biological control, mulberry, root exudates, *Ralstonia pseudosolanacearum*

## Abstract

Bacterial wilt is a significant soil-borne disease that poses a threat to mulberry production yield and quality of agricultural production worldwide. However, the disease resistance mechanisms dependent on root exudates are not well understood. In this present study, we investigated the antibacterial mechanisms of the main active substances (erucamide, oleamide, and camphor bromide) present in mulberry root exudates (MRE) against *Ralstonia pseudosolanacearum* (*Rp*), the causal agent of bacterial wilt. Our findings revealed that these three active substances inhibited the growth activity of *Rp* by affecting the cell morphology and extracellular polysaccharide content, as well as triggering a burst of reactive oxygen species. The active substances induced oxidative stress, leading to a decrease in *Rp* growth. Additionally, the expression levels of key genes in the hrp gene cluster (*hrpB*, *hrpX*, and *hrpF*) and other virulence-related genes (such as *ripAW*, *ripAE*, *Rs5-4819*, *Rs5-4374*, *ace*, *egl3*, and *pehB*) were significantly reduced upon treatment with the active substances. Further pathogenicity experiments demonstrated that root exudates (at a concentration of 1.5 mg·mL^−1^) delayed or slowed down the occurrence of bacterial wilt in mulberry. These findings provide valuable insight into the antimicrobial mechanisms of MRE against *Rp* and lay a theoretical foundation for the development and application of biocontrol agents to control mulberry bacterial wilt.

## 1. Introduction

The mulberry tree (*Morus alba* L.), an economically and ecologically significant plant species with a global presence, serves various roles [1]. Notably, it is a vital resource for the silk industry as the primary food source for silkworms [1]. Beyond this traditional application, its leaves are also utilized as feed supplements for livestock and poultry due to their rich protein content [1,2]. Furthermore, mulberry’s high nutritional value and bioactive components make it a sought-after ingredient in human foods and in various formulations of traditional Chinese medicine, thus underlining its multifaceted importance across sectors. However, the production of mulberry is threatened by numerous diseases, with bacterial wilt caused by *Rp* being one of the most destructive soil-borne diseases that limits healthy production [3,4]. The initial outbreak of mulberry bacterial wilt was documented in 1969, originating from Shunde City within Guangdong province, China. Since that pivotal discovery, the disease has progressively disseminated to encompass the majority of mulberry cultivation regions across Guangdong and beyond, affecting various parts of the country [5]. Currently, the prevention and control of bacterial wilt mainly rely on preventive measures and chemical pesticides, which can lead to environmental pollution. The lack of effective control methods for bacterial wilt poses a serious threat to the sustainable development of the sericulture industry. Therefore, it is crucial to explore new ecological bacteriostatic agents for the prevention and control of bacterial wilt in mulberry. This will help to reduce the reliance on chemical pesticides and promote sustainable development in the sericulture industry.

Plant root exudates play a pivotal role in mediating plant growth and development, as well as fostering intricate interactions between the plant roots and the surrounding rhizosphere’s physicochemical attributes and biological components [6]. Root exudates (RE) are a diverse group of compounds released by plant roots into the surrounding environment that encompass both primary metabolites such as amino acids, sugars, and other essential organic compounds, alongside an array of secondary metabolites, including flavonoids and coumarins [7,8]. Root exudates act as a pivotal mediator in the intricate web of interactions among plants, soil components, and pathogenic microorganisms. They exert a direct influence on the growth, proliferation, and virulence of soil-dwelling pathogens, thereby significantly impacting the incidence and progression of soil-borne diseases [9]. For example, cinnamic acid, berberine, and myristicin in tobacco root exudates can induce the chemotaxis of *R. solanacearum*, stimulate biofilm formation, and promote the occurrence of bacterial wilt in tobacco [10]. Fatty acid amides in plant root exudates also play a role in the early development of seedlings and the interaction between plants and microorganisms [11,12]. Oleamide and erucamide are involved in the interaction between plants and microorganisms, stimulating the nitrogen metabolism of rhizobacteria [13]. As a perennial woody plant, mulberry release various root exudates into the surrounding soil during its growth process. After stem and leaf pruning of mulberry seedlings, the secretion of amino acids in root exudates increases, and mulberry root exudates promote the germination of fungal spores, the growth of hyphae, the accumulation of motile spores, and the enhancement of pathogenicity [14]. However, there have been few reports on the specific components of mulberry root exudates and their interactions with pathogens. Therefore, studying the composition of mulberry root exudates and their effects on the growth of *Rp* is of great theoretical and practical significance for understanding and coordinating the interaction between mulberry and *Rp*.

In our preceding research, we successfully gathered and purified root exudates from hydroponically cultivated “Feng Chi” mulberry seedlings. Subsequently, these exudates were subjected to Gas Chromatography–Mass Spectrometry (GC–MS) for meticulous identification and detailed compositional analysis under different pH conditions. It is worth noting that previous research has demonstrated the suppressive impact of these root exudates compounds on the proliferation of *Rp* [15]. The pathogenicity of *Rp* is largely attributed to various factors, including EPS for colonization and protection, T3SS for delivering virulence effectors into host cells, cell wall-degrading enzymes to facilitate infection, and motility for spreading within the plant. The expression of T3SS-related genes is regulated by transcription factors like HrpB, HrpG, PrhG, and PhcA, with PhcA playing a central role in coordinating the expression of virulence factors while modulating EPS production, cellulase activity, swimming motility, and type III secretion system (T3SS) gene expression [16,17]. The hrp (hypersensitive response and pathogenicity) gene cluster in *Ralstonia* encodes the type III secretion system, which secretes effector proteins and delivers them into the cytoplasm of host plant cells [18]. Studies have also shown that excessive accumulation of reactive oxygen species (ROS), such as exogenous reactive oxygen species generated after disinfection and antibiotic treatment, can cause oxidative stress, leading to oxidative damage and affecting bacterial activity [19]. Plant-derived compounds (PDCs), particularly those secreted by roots as part of their defense mechanism, can exhibit potent antibacterial properties. For example, compound 7-methoxycoumarin has antibacterial effects on the growth of *R. solanacearum* [20]. Plants have evolved a complex array of defense mechanisms to protect themselves against pathogens. One such strategy involves the production and secretion of secondary metabolites by plant roots. These compounds are not directly involved in primary metabolic processes like growth and reproduction but play crucial roles in plant survival. Compounds include indoles, phenols, terpenes, flavonoids, benzoxazinones, and coumarins, all of which exhibit strong antibacterial properties [21]. Hydroxycinnamic acid produced by plants affects the virulence and colonization ability of *Ralstonia* [22]. Umbelliferone reduces biofilm formation and regulates the transcription of T3SS regulatory factors and effectors in *R. solanacearum* [23]. Protocatechualdehyde (PCA), a naturally occurring polyphenolic constituent derived from the roots of *Salvia miltiorrhiza*, a widely used herb in traditional Chinese medicine, has been reported to significantly inhibit the growth of *R. solanacearum*, disrupt cell structure and morphology, and inhibit biofilm formation [24]. 4-Methoxycinnamic acid, benzoic acid, and trans-4-hydroxycinnamic acid isoxime have been shown to inhibit the HrpX/HrpY two-component signal transduction and biofilm formation in plant pathogens [25]. MRE as substances derived from plant roots have been proven to inhibit the growth of *Rp* in mulberry and alter its bacterial morphology through active substances such as erucamide, oleamide, and camphor bromide.

In this study, we investigated the antibacterial mechanism of erucamide, oleamide, and camphor bromide, which are active substances found in MRE. We examined the impacts of these compounds on the morphology of *Rp*, the generation of reactive oxygen species (ROS), and the production of extracellular polysaccharides (EPS). ROS are known to play a role in bacterial stress responses and can affect bacterial growth and survival. EPS are important virulence factors in *Rp* and contribute to its pathogenicity [9]. We measured the generation of ROS and EPS in *Rp* after treatment with the active substances. To further understand the molecular mechanisms underlying the antibacterial effects of these active substances, we measured the expression levels of relevant virulence genes in *Rp* using real-time fluorescence quantitative PCR. This allowed us to assess the impact of erucamide, oleamide, and camphor bromide on the regulation of genes associated with pathogenicity, such as those involved in the type III secretion system (T3SS) and effector proteins (T3Es).

To our knowledge, there are few studies using root exudates to control mulberry diseases, particularly bacterial wilt caused by *Rp*. This present study was designed to delve into the antibacterial properties and underlying mechanisms of mulberry root exudates against *Rp*. We employed scanning electron microscopy (SEM) to visualize any morphological alterations in *Rp* cells resulting from exposure to these exudates. Active substances induce a burst of reactive oxygen, triggering oxidative stress and exopolysaccharides content, inhibiting the growth of *Rp*. This study provides valuable insights into the inhibitory mechanisms of MRE against *Rp*, thereby establishing a theoretical foundation for utilizing root exudates as a natural and sustainable strategy to control mulberry bacterial wilt.

## 2. Results

### 2.1. The Effect of Active Substances in Root Exudates on the Growth of Rp

**Figure 1:**
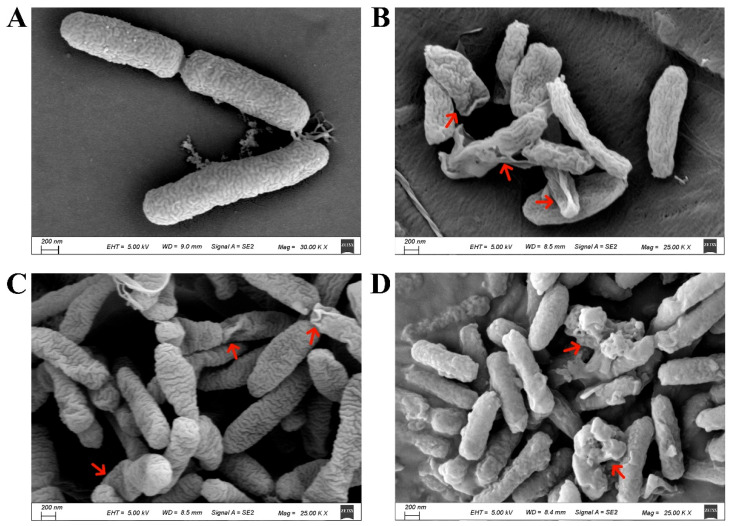
Scanning electron microscopy analysis of *Ralstonia pseudosolanacearum* following the addition of active substances from mulberry root exudates. The control group (**A**) consisted of bacteria that were not subjected to any treatment. In contrast, (**B**–**D**) display bacterial populations treated with different substances: erucamide (**B**), oleamide (**C**), camphor bromide (**D**). Notably, red arrows denote the presence of cells exhibiting anomalous morphologies in each respective treatment condition.

Root exudates play an important mediating role between plants and soil microorganisms, rhizosphere organisms, and the soil environment [26]. Previous studies have determined the composition of mulberry root exudates under neutral, acidic, and alkaline extraction conditions [15]. The total ion chromatograms (TIC) of mulberry seedling root exudates under different pH extraction conditions were analyzed using GC–MS (Supplementary Figure S2). The results showed that the retention times of the components in the root exudates were relatively similar under alkaline, acidic, and neutral extraction conditions, but the relative peak intensities differed significantly. A total of 137 substances were identified, and among them, the amide substances had the highest relative content, accounting for 42.6% of the total [15]. Camphor bromide was only present in the neutral component of the root exudates and had a relatively high relative content of 18.33% (Supplementary Figure S2). To discern the influence of principal active substances in root exudates on the colony morphology and proliferation dynamics of *Rp*, scanning electron microscopy (SEM) was used to observe the morphological changes of MRS-5 after treatment with three different active substances. In the solvent control group, the surface structure of the bacterial cells appeared intact, and their morphology was full, showing no significant differences compared to the normal growth conditions of MRS-5 (Figure 1A). However, compared to the solvent control group, the MRS-5 strains treated with 1.5 mg/mL of erucamide, oleamide, and camphor bromide exhibited severe deformation in their morphology. The bacterial cells appeared flattened and wrinkled, as indicated by the red arrows (Figure 1B,C). Among them, the oleamide-treated group showed a blurry surface of the bacterial cells, with the accumulation of dissolved substances around the cells (Figure 1D).

### 2.2. The Effect of Root Exudates on the Extracellular Polysaccharide Production of Rp

**Figure 2:**
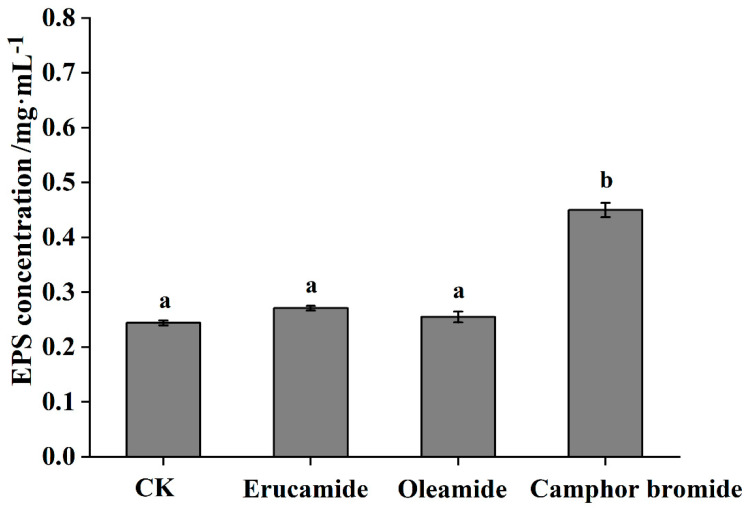
The effect of three active substances in root exudates on the synthesis of extracellular polysaccharides by *Ralstonia pseudosolanacearum*. The control group, designated as CK, was treated with ethanol solvent alone, while the concentration of each tested active substances was set at 1.5 mg·mL^−1^. Post hoc comparisons based on Duncan’s multiple range test after a one-way analysis of variance showing means for treatment groups differing significantly (*p* < 0.05); same letters indicate no significant difference.

Extracellular polysaccharides (EPS) constitute a key pathogenic factor that can instigate vascular blockages and impair and disrupt the flow of water within the plant system, culminating in a characteristic wilt [27,28]. EPS not only promotes the systemic colonization of *R. solanacearum* within the host but also severely blocks the host vascular tissue, leading to plant dehydration and death. It is crucial for the pathogenic process of *R. solanacearum*. In our previous study, When the concentration of the compounds was 1.5 mg·mL^−1^, both oleamide and camphor bromide exhibited an antibacterial rate of over 90%, while erucic acid amide showed an antibacterial rate of over 80% [15]. In this study, treatment with 1.5 mg/mL of erucamide and oleamide did not significantly affect the EPS content of *Rp*. However, treatment with the same concentration of camphor bromide resulted in a significant increase in the EPS content of *Rp* (Figure 2). This suggests the increased permeability of the cell membrane of *Rp* strains MRS-5 after camphor bromide treatment.

### 2.3. The Effect of Root Exudates on the Burst of Reactive Oxygen Species in Rp

**Figure 3:**
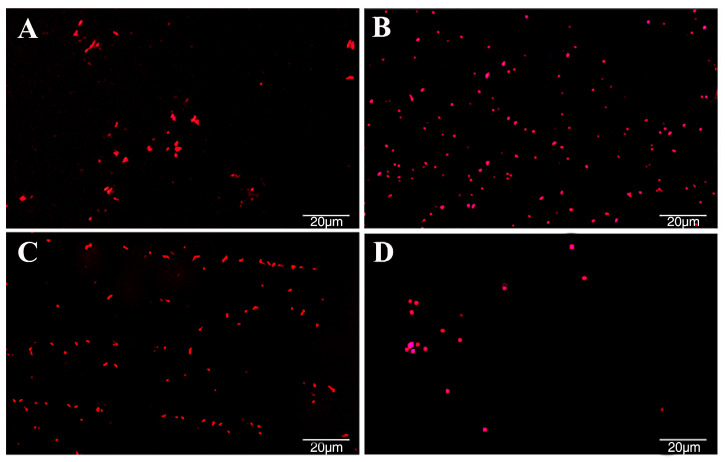
Investigation of the impact of three active substances found in root exudates on the release of reactive oxygen species (ROS). A DCF-fluorescent imaging technique was captured to visually demonstrate the effect of these substances at a concentration of 1.5 mg·mL^−1^, where the intensity of ROS production is depicted by bright fluorescent dots within the images. *Ralstonia pseudosolanacearum* was subjected to treatment with erucamide (**A**), oleamide (**B**), camphor bromide (**C**), while an ethanol solvent served as the control (**D**). The fluorescence signals were captured using fluorescence microscopy (equipped with a 488 nm filter; OLYMPUS IX-71 microscope, Tokyo, Japan) [29].

Excessive reactive oxygen species (ROS) within bacterial cells can cause oxidative stress, leading to oxidative damage and even affecting their activity [21]. The antibacterial strength of different active substances against *Rp* in mulberry was evaluated by observing and measuring the production of ROS at the same concentration. The results of this study showed that at a concentration of 1.5 mg·mL^−1^, erucic acid amide, oleamide, and camphor bromide all induced a significant generation of ROS within *Rp* cells, as indicated by the red punctate material in the figure (Figure 3A–C). Among them, oleamide had the most pronounced effect on ROS generation in *Rp*, followed by camphor bromide (Figure 3B,C). Compared to the solvent control group (Figure 3D), *Rp* treated with erucic acid amide, oleamide, and camphor bromide showed a significant increase in ROS within the same area, indicating that the three active substances caused oxidative damage to *Rp*, thereby affecting bacterial growth.

### 2.4. The Effect of the Main Active Substances in Root Exudates on the Expression of Pathogenicity-Related Genes in Rp

**Figure 4:**
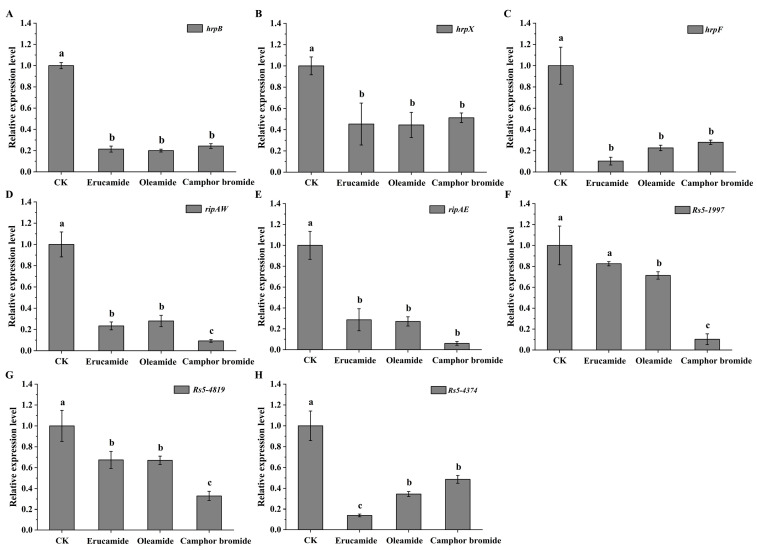
Analysis of the expression pattern of type III secretion system-related virulence genes in *Ralstonia pseudosolanacearum* upon treatment with three active substances. The graphic illustrates the effects of erucamide, oleamide, and camphor bromide (1.5 mg·mL^−1^) on the transcription levels of several key genes associated with the hrp gene cluster: *hrpB* (**A**), *hrpX* (**B**), *hrpF* (**C**). Additionally, it demonstrates their influence on the expression of T3Es related genes, including *ripAW* (**D**), *ripAE* (**E**), *Rs5-1997* (**F**), *Rs5-4819* (**G**), and *Rs5-4374* (**H**) in *R. pseudosolanacearum*. The ethanol solvent control (CK) was utilized as a reference. Post hoc comparisons based on Duncan’s multiple range test after a one-way analysis of variance showing means for treatment groups differing significantly (*p* < 0.05); same letters indicate no significant difference.

The T3SS plays a central role in the pathogenic mechanisms of bacterial pathogens in many animals and plants [30,31]. Previous studies have shown that the *hrp* genes of *Rp* are involved in mediating plant hypersensitive response (HR). Additionally, this gene cluster regulates the transcriptional expression of the type III secretion system and effector proteins, thereby determining the pathogenicity of *Rp* [32]. In this study, the changes in the expression levels of T3SS-related virulence genes in *Rp* strain MRS-5 were determined after treatment with three active substances present in root exudates. The expression of the main regulatory gene *hrpB*, which controls the type III secretion system, was significantly downregulated in *Rp* after treatment with erucamide, oleamide, and camphor bromide, showing a five-fold decrease compared to the solvent control group (Figure 4A). The expression of *hrpX*, a gene associated with flagellar assembly, and *hrpF*, a gene associated with translocator proteins, were both significantly downregulated in the active substance-treated MRS-5 strain (Figure 4B,C). To further investigate the impact of active substances on the gene expression levels of T3Es (RipAW, RipAE, Rs5-1997, Rs5-4819, and Rs5-4374) in *Rp* infecting mulberry, the expression of these T3Es was measured after treatment with the active substances. The results showed that erucamide, oleamide, and camphor bromide led to a significant downregulation of T3Es gene expression (Figure 4D–H). Among the treatment groups, camphor bromide exhibited a more pronounced inhibitory effect on the gene expression of *ripAW*, *Rs5-1997*, and *Rs5-4819* (Figure 4D,F,G), while erucamide showed more significant inhibition of *Rs5-4374* gene expression (Figure 4H). However, after treatment with erucamide, the gene expression of *Rs5-1997* decreased but without significant difference (Figure 4F).

**Figure 5:**
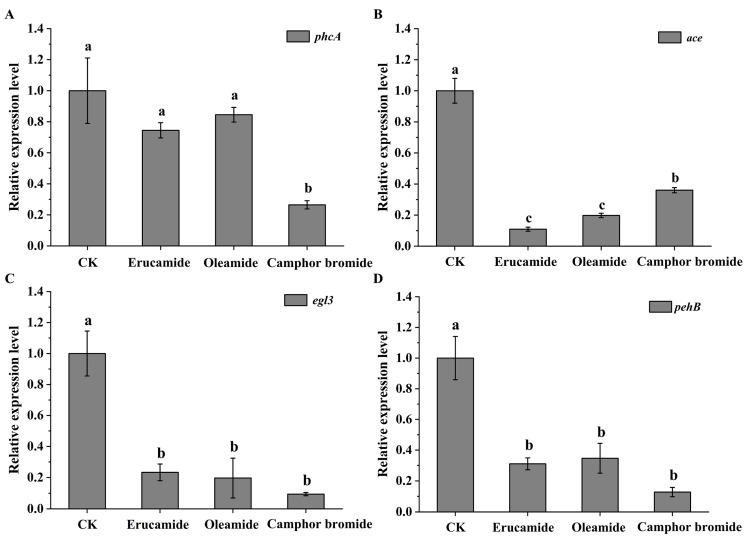
Analysis of the expression patterns of virulence-related genes in *Ralstonia pseudosolanacearum* treated with three active substances. This figure presents the effect of erucamide, oleamide, and camphor bromide (1.5 mg·mL^−1^) on the expression levels of hrp gene cluster that are crucial for virulence: *phcA* (**A**), *ace* (**B**), *egl3* (**C**), and *pehB* (**D**) in *Rp*. The ethanol solvent control (CK) was employed as a reference. Post hoc comparisons based on Duncan’s multiple range test after one-way analysis of variance showing means for treatment groups differing significantly (*p* < 0.05); same letters indicate no significant difference.

The regulatory mechanism of virulence factors in *Rp* is complex. To further investigate the impact of active substances on the molecular regulation mechanism of *Rp* in mulberry, this experiment measured the changes in the expression levels of the transcriptional regulatory factor phcA, which belongs to the LysR family, in *Rp*. Based on real-time quantitative PCR experiments, it was found that the expression level of phcA in the MRS-5 strain significantly decreased after treatment with camphor bromide. However, although erucamide and oleamide downregulated the expression of phcA, there was no significant difference (Figure 5A). This study further extended to evaluate the effects of the three active compounds on the expression patterns of additional virulence-associated genes, including the acid dehydrogenase-coding gene *ace*, the cellulase-related gene *egl*, and the pectinase-governing gene *pehB*. Compared to the solvent control, erucamide, oleamide, and camphor bromide all led to a significant decrease in the expression levels of *ace*, *egl3*, and *pehB* (Figure 5B–D).

### 2.5. The Effects of Active Substances in Root Exudates on the Pathogenicity of Rp and the Growth of Mulberry Seedlings

**Figure 6:**
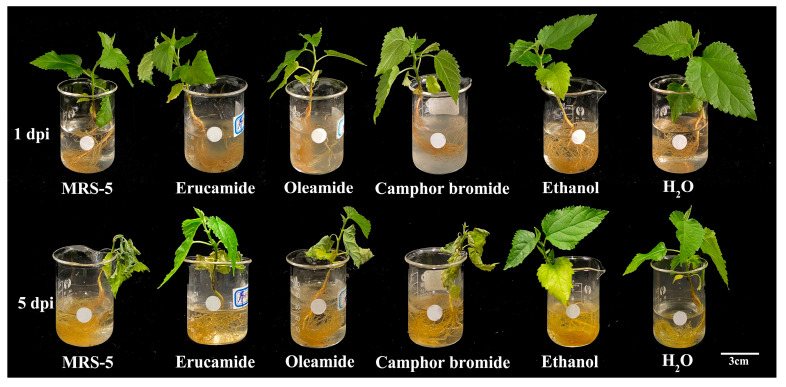
Influence of three active substances on the pathogenicity of *Ralstonia pseudosolanacearum*. The figure depicts the pathogenic effects under different conditions as follows: MRS-5 indicates where only *Rp* was inoculated; erucamide treatment, where *Rp* was treated with erucamide; oleamide treatment, in which *Rp* and oleamide were combined; camphor bromide represents treatment with *Rp* along with camphor bromide. The sterile water served as a blank control, and ethanol acted as the solvent control. In all substance treatments, the active substances were used at a concentration of 1.5 mg·mL^−1^. Evaluate differences in pathogenicity based on leaf wilting. dpi denotes days post infection with *Rp* for monitoring disease progression.

This study shows that three active substances have inhibitory effects on the pathogenic determinants of *Rp* MRS-5 in mulberry. To clarify the effect of root exudates on the pathogenicity of *Rp*, we use the root inoculation method to determine the pathogenicity of mulberry seedlings. In the control group (only added MRS-5), obvious wilting symptoms appeared in the mulberry seedlings after 5 days post-inoculation (dpi). However, the pathogenicity of MRS-5 was weakened after treatment of erucamide, oleamide, and camphor bromide with 1.5 mg·mL^−1^ separately, especially treated by erucamide with only a few curling leaves observed (Figure 6). Conversely, mulberry seedlings treated with oleamide and camphor bromide exhibited noticeable wilting symptoms even after MRS-5 infection (Figure 6). This observation suggests that the three active substances present in mulberry root exudates have a discernible influence on the pathogenicity of *Rp*.

**Figure 7:**
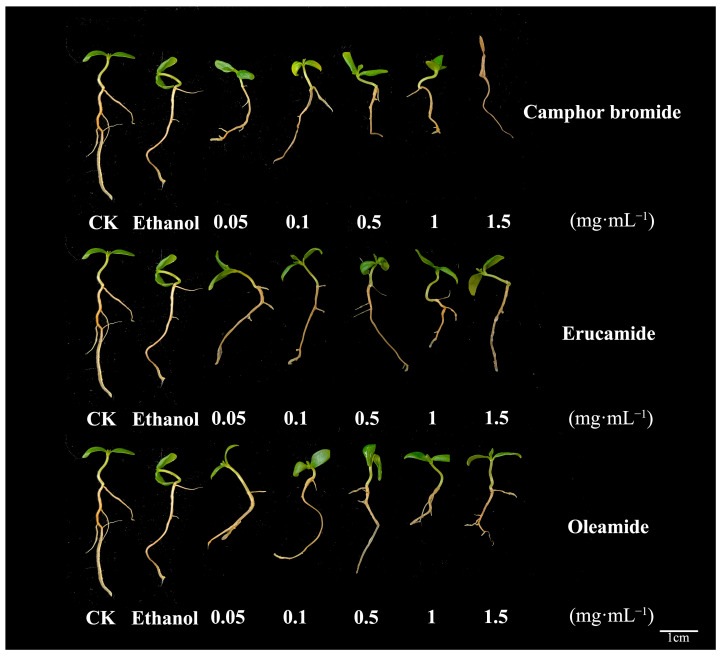
Assessment of the impact of three active substances on the growth performance of mulberry seedlings. This figure illustrates the growth status of mulberry seedlings following a 7-day treatment with three active substances at varying concentrations (0.05, 0.1, 0.5, 1.0, and 1.5 mg·mL^−1^). The sterile water control (CK) was employed as the reference to gauge natural growth conditions, while ethanol served as the solvent control to account for any potential effects from the delivery medium. Root length as an indicator to measure the effect of active substances on the growth of mulberry seedlings.

In order to examine the potential effects of these active substances on seedling growth, mulberry seedlings were subjected to various treatment concentrations (0.05, 0.1, 0.5, 1.0, and 1.5 mg·mL^−1^) of the active substances. Subsequently, their growth patterns were meticulously observed over a period of 7 days following the treatments. The impact of different active substance treatments on the growth of mulberry seedlings was evaluated by measuring the differences in root length of mulberry seedlings. The results showed that camphor bromide inhibited the root growth and development of mulberry seedlings to some extent, and high concentrations led to seedling death. Oleamide also had an inhibitory effect on the root growth of mulberry seedlings at high concentrations. However, erucamide did not show a significant impact on the growth of mulberry seedlings under the experimental conditions (Figure 7). In conclusion, all three active substances were able to reduce the pathogenic determinants of MRS-5, but oleamide and camphor bromide affected the root growth and development of mulberry itself at high concentrations, while erucamide did not exhibit significant inhibitory effects. Therefore, erucamide could be considered as a potential biocontrol resource for the prevention and treatment of mulberry bacterial wilt.

## 3. Discussion

Root exudates represent the chemical substances that plants discharge from their roots into the surrounding soil environment [33]. Plant root exudates, as important signaling substances, participate in the interaction process between microorganisms and host plants [34]. The quantity and chemical composition of root exudates are subject to spatial and temporal fluctuations, thereby giving rise to a dynamic interplay between plant roots and their immediate soil environment [33]. At the individual plant level, root exudation is influenced by an array of factors such as plant genetics, development stage, root system architecture, and nutritional condition [33]. In recent years, many components of plant root exudates have been identified in different plants, including *Arabidopsis*, soybean, rice, etc. However, current research mainly focuses on herbaceous or shrub plants, with only a few woody plant species such as apple, peach, and jujube being studied. Apple seedling root exudates were found to primarily contain organic acids, ethylene glycol, esters, and phenolic derivatives through GC–MS detection; peach tree root exudates contained phenolic acids and phenolic derivatives, as well as two unknown compounds; jujube root exudates did not contain any phenolic acid substances [35]. Our previous studies have reported that in mulberry root exudate composition in different pH extraction conditions, a total of 137 substances were obtained [15]. The main components of root exudates from mulberry seedlings under hydroponic conditions were determined, laying a theoretical foundation for further research on the role of root exudates in the growth process of mulberry.

Bacterial wilt is a plant disease caused by various types of bacteria, with the most common being *R. solanacearum*. This disease affects many important economic crops worldwide, including tomatoes, potatoes, tobacco, and bananas, among others [36]. Mulberry bacterial wilt constitutes a grave and devastating soil-borne disease, instigated by a multifaceted and diverse array of pathogenic bacteria that pose a considerable menace to global mulberry cultivation practices [3,15]. *R. solanacearum* has the ability to persist in soil for extended periods outside host plants, thereby presenting formidable obstacles to effective control measures. The use of traditional chemical pesticides can cause harm to the environment and human health. Consequently, there is an urgent necessity to develop innovative ecological management strategies for the efficient control of bacterial wilt. Plants contain abundant antimicrobial substances, which are easily biodegradable, environmentally friendly, and cost-effective [37,38]. The pathogenic interaction between *R. solanacearum* and its host plants is characterized by a complex interplay of pathogenesis mechanisms, which can be broadly categorized into three critical phases: initial contact with the host plant, attachment to the plant root surface, and subsequent invasion of the root cortex followed by colonization within the xylem’s parenchymatous tissues [28]. This intricate infection process is orchestrated by a myriad of virulence factors working in concert, such as the Type III secretion system and effector proteins, exopolysaccharides, and motility capabilities, among others. Previous research has reported that ginger extract demonstrates antibacterial properties against a wide range of microorganisms, including both Gram-positive and Gram-negative bacteria, aquatic pathogens, and food-borne pathogens [39]. Consistently, it has been demonstrated that ginger extract possesses potent antibacterial properties against *R. solanacearum*, and the bactericidal effect was observed to be dose-dependent [28]. It was reported that phenolic acids in plant root exudates have inhibitory effects on soilborne pathogens, indicating the feasibility of using plant-derived compounds to prevent bacterial wilt [40]. Methyl gallate (MG) has been shown to significantly inhibit the growth of the bacterial pathogen causing bacterial wilt, as it disrupts the cell structure of the pathogen and inhibits protein synthesis and succinate dehydrogenase (SDH) activity [41]. Compounds such as lansoprazole B, thymol, and palm oil also exhibit antimicrobial activity against the bacterial pathogen causing bacterial wilt [38]. Phenolic acid compounds were isolated and characterized from the leaves of Artemisia herba-alba, following which, the antibacterial potency of both the extracts and the identified phenolic acid was tested against a selection of bacterial strains [42]. Additionally, there are reports indicating that phenolic acids, as well as alkaloids, exhibit efficacious antimicrobial activity against a variety of bacteria, including *Staphylococcus aureus*, *Enterococcus faecalis*, *Bacillus subtilis*, *Moraxella catarrhalis*, and *Escherichia coli* [43,44]. In addition, we also determined the antibacterial activity of these three active substances against mulberry anthracnose and mulberry blight. The preliminary results indicate that the antibacterial activity can reach over 80% (data has not been published yet). Although the antimicrobial activity of mulberry root exudates has already been detected [15], the underlying antimicrobial mechanism remains unclear. In our previous study, a total of 137 compounds were analyzed, and among these, amide substances and camphor bromide emerged as the two most abundant components. Specifically, amide substances accounted for 42.6% of the total content in the sample mixture, meanwhile, camphor bromide was found to contribute to approximately 18.33% of the overall content [15]. Three mulberry root exudate active substances can significantly inhibit the formation of biofilms in *Rp*. In addition, the three active substances inhibit the motility of *Rp*, especially the most significant inhibitory effect of camphor bromide.

Extracellular polysaccharides are an important virulence factor of *Ralstonia*, which is involved in pathogen adhesion, biofilm formation, and immune escape [45]. This study determined the antibacterial mechanism of three main active substances against mulberry wilt disease. Three active substances can inhibit the growth activity of *Rp* by affecting the cell morphology and extracellular polysaccharide content (Figure 2). Reactive oxygen species have a dual role in living organisms, serving as signaling molecules to participate in normal physiological processes and potentially triggering oxidative stress leading to cell damage [46,47]. In this study, we found that active substances induce a burst of reactive oxygen, triggering oxidative stress and inhibiting the growth of *Rp* (Figure 3). The antibacterial activities of ginger extract against the ginger bacterial wilt pathogen *R. solanacearum* [28]. It has been shown that maintaining a lower concentration of root exudates can stimulate an increase in ginseng rhizome biomass and enhance the accumulation of ginsenosides within the rhizomes [48]. The Type III Secretion System (T3SS) of *R. solanacearum* is one of its most important virulence factors [49]. The T3SS is capable of directly injecting effector proteins into host cells, interfering with the host’s physiological processes and promoting pathogen infection and colonization [50]. Multiple T3SS effector proteins encoded by *R. solanacearum* have been identified, such as RipA through RipW, which play crucial roles in disrupting plant defense responses and establishing effective infections [51]. The type III secretion system genes, particularly those within the hrp cluster, along with the popA gene, play a crucial role in the regulation and execution of *R. solanacearum* infection processes [52]. It was found that 7-methoxycoumarin significantly inhibits the expression of hrpG and popA [22]. Hydroxycoumarins reduce the pathogenicity of bacterial wilt by inhibiting T3SS and the formation of biofilms [53]. In this study, active substances led to a significant decrease in the expression levels of *hrpB*, *hrpX*, and *hrpF* genes in the hrp gene cluster, as well as the expression of other virulence-related genes such as *ripAW*, *ripAE*, *Rs5-4819*, *Rs5-4374*, *ace*, *egl3*, and *pehB* (Figure 4 and Figure 5). Additional pathogenicity experiments demonstrated that the presence of root exudates effectively retarded or mitigated the onset and progression of bacterial wilt in mulberry (Figure 6).

The root system of mulberry may secrete a series of compounds with antibacterial activity, such as phenols, flavonoids, and alkaloids. These compounds may directly interfere with the cell membrane stability, protein synthesis, or DNA replication of *R. solanacearum*, thereby inhibiting its growth and reproduction. Some root exudates can induce plants to produce defense-related substances such as ROS, PR proteins, and phytoalexins. These substances may directly kill pathogens or enhance the plant’s resistance to pathogen attacks. The inhibitory mechanism of mulberry root exudates on *Rp* may involve multiple aspects, requiring further experimental research to reveal its detailed molecular mechanisms and action pathways. Moreover, these findings provide an important theoretical basis and practical guidance for the development of new biological control strategies.

## 4. Materials and Methods

### 4.1. Plant Materials and Bacterial Strains

Mulberry variety Fengchi seeds were provided by the Mulberry Research Office of our college. In this research, we employed the *Rp* strain MRS-5 (GDMCC1.3250), initially isolated in our lab from diseased mulberry plants located in Luogang, Guangdong Province. The cultivation process involved growing MRS-5 on a casamino acid–peptone–glucose (CPG) agar plate [53], with incubation at 30 °C for 72 h [48]. Subsequently, a single colony was selected and inoculated into 150 mL of CPG liquid medium. This culture was then grown at a constant temperature of 30 °C with agitation at 180 rpm for approximately 16 to 18 h, thereby yielding the *Rp* fermentation broth.

### 4.2. GC/MS Analysis of Mulberry Root Exudates

Following a 14-day period of meticulous mulberry cultivation, a volume of 600 mL of the culture medium was collected and filtered through an organic filter membrane with a pore size of 0.22 µM (Supplementary Figure S1). The resulting filtrate was extracted using ethyl acetate. The three different components obtained from the extraction were concentrated to 5 mL using a rotary evaporator (45 °C, 50 rpm) and analyzed using a GC/MS (Thermo Trace-1300 ISQ (ThermoFisher, Waltham, MA, USA)) gas chromatography–mass spectrometry instrument [34]. The chromatographic column employed in this study was a DB-5MS column (30 m × 0.25 mm × 0.25 µm). The ionization source used was the Electron Ionization (EI^+^) mode. The temperature was set to 250 °C for both the ion source and inlet. The column temperature was programmed to increase from 80 °C to 200 °C at a gradual rate of 15 °C·min^−1^, and the temperature was further increased to reach 250 °C at a rate of 1.5 °C·min^−1^. Throughout the process, helium served as the carrier gas maintained at a consistent flow rate of 1 mL·min^−1^. The injection volume for each sample was precisely set to 1 μL. The relative content of each component in the GC–MS spectrum was calculated using the peak area normalization method, and the composition of the components was determined by automatically searching for each chromatographic peak in the NIST 2014 mass spectral database and combining it with manual analysis.

### 4.3. Antimicrobial Activity of Mulberry Root Exudates

The Oxford cup method was used to investigate the antimicrobial ability of the mulberry root exudates [28]. The assessment of the inhibition ability of mulberry root exudates against *Rp* was evaluated in 1.5 mL test tubes. Erucamide, oleamide, and camphor bromide were all purchased from Aladdin Reagent Company. Briefly, 20 μL of different concentrations of erucamide, oleamide, and camphor bromide (concentration gradient of 0.05, 0.1, 0.3, 0.5, 1, and 1.5 mg·mL^−1^) were added to tubes, followed by the addition of 140 μL of CPG liquid medium and 40 μL of bacterial suspension with an optical density of OD_600_ of 0.6, up to 200 μL of mixed solution; equivalent ethanol was taken as the solvent control, and 20 μL of CPG medium was used as the blank control. After adding the root exudate components, the mixed solution was measured for OD_600_ value and was recorded as T0. The culture mixture was incubated in a shaker at 180 rpm at 28 °C. The OD_600_ was measured precisely after a 24 h incubation recorded as TF. For each treatment, three technical replicates were performed to ensure accuracy and consistency. The inhibitory rate was then calculated using the following formula:

$$Inhibition(\%)=\left(1-\frac{{TF}_{sample}-{T0}_{sample}}{{TF}_{blank}-{T0}_{blank}}\right)\times 100$$

### 4.4. Scanning Electron Microscopy Analysis

For scanning electron microscope (SEM) analysis to examine the growth morphology of *Rp*, cell slides were placed in a 12-well plate and CPG medium and MRS-5 bacterial suspension (OD_600_ = 0.6) were added. The plate was then incubated at 28 °C for 12 h [28]. After incubation, the slides were treated with erucamide, oleamide, and camphor bromide at a concentration of 1.5 mg/mL (with anhydrous ethanol as the solvent control), followed by continued incubation for 8 h. The bacteria cells present in the slides were dehydrated with a gradient of alcohol at different concentrations, followed by drying, and then coated with gold using an SCD 500 ion sputter coater. The morphological changes of *Rp* cells were observed using SEM (equipped with 5.00 kV EHT, SE2 signal, GeminiSEM 300, Carl Zeiss, Oberkochen, Germany).

### 4.5. Extracellular Polysaccharide Content Determination

The content of extracellular polysaccharides from the *Rp* was determined using the phenol-sulfuric acid method with aqueous alcohol precipitation [54]. In this process, 5 mL of the bacterial culture was subjected to centrifugation at 10,000 rpm for 10 min. Subsequently, the pellet was treated overnight with 5 mL of erucamide, oleamide, and camphor bromide (1.5 mg/mL). The supernatant was collected and mixed with four times the volume of anhydrous ethanol, followed by overnight precipitation of extracellular polysaccharides at 4 °C. After centrifugation for 10 min, the supernatant was discarded, and the pellet containing the polysaccharides was dissolved in water, thus yielding a crude polysaccharide solution. A glucose standard curve was prepared using glucose as a control. Then, 200 μL of different treated crude polysaccharide solutions, 100 μL of 4% phenol solution, and 500 μL of concentrated sulfuric acid were mixed. After 15 min, the absorbance was measured at 490 nm. The extracellular polysaccharide content in the treated groups was calculated based on the standard curve [55]. Ethanol served as a solvent control, and each treatment was repeated three times [53].

### 4.6. Measurement of Reactive Oxygen Species

The measurement of intracellular reactive oxygen species levels was detected using the 2′, 7′-dichlorofluoresceindiacetate (DCFH-DA) fluorescence probe (Beyotime, Nanjing, China) [56]. The *Rp* was cultured until OD_600_ reached 0.6, and erucamide, oleamide, and camphor bromide were separately added at a concentration of 1 mg·mL^−1^. The control group was subjected to treatment with an equal volume of ethanol. Subsequently, the culture cells were allowed to proceed for an additional 8 h. The bacterial suspension was centrifuged at 8000× *g* rpm, 25 °C, for 2 min to remove the supernatant. The pellet was washed gently three times with sterile water. Then, the diluted 10 mM DCFH-DA was added at a ratio of 1:1000, and the samples were incubated at 30 °C in the dark for 30 min. After removing DCFH-DA, the samples were thoroughly washed with sterile water. Finally, the fluorescence of the samples was measured using a confocal laser scanning microscope. Each treatment was repeated three times.

### 4.7. Gene Expression Analysis

The effect of root exudates on the expression levels of virulence-related genes of *Rp* was assessed through quantitative real-time PCR, following a previously established protocol [57]. At 24 h post-treatment, total RNA was extracted using the RNAiso Plus reagent (Takara, San Jose, CA, USA) to capture any changes in gene expression. The quantity and quality of the isolated RNA were meticulously examined utilizing a NanoDrop 8000 (Thermo Scientific, Waltham, MA, USA). cDNA synthesis was performed with equal amounts of total RNA from each sample, employing the HiScript^®^ II 1st Strand cDNA Synthesis Kit (Vazyme, Nanjing, China). For the RT-qPCR experiments, the ABI QuantStudio 3 Real-Time PCR System (Thermo Fisher) was used, coupled with ChamQ™ Universal SYBR^®^ qPCR Master Mix (Vazyme). All RT-qPCR experiments were performed using specific primers (Table S1) with the constitutively expressed *16S rRNA* gene serving as a reference, and relative gene expression was calculated adopting the comparative 2^−ΔΔCT^ method.

### 4.8. Controlling Bacterial Wilt by Mulberry Root Exudates

The effectiveness of root exudates in controlling mulberry bacterial wilt was ascertained using a hydroponic root invasion assay. To inoculate the MRS-5 strain, a root irrigation method was used [49]. The MRS-5 bacteria were cultured overnight at 28 °C in CPG liquid medium to ensure adequate growth. Bacterial cells were collected by centrifugation, washed with sterile water, and adjusted to a final OD_600_ of 0.5. For treatment, the bacterial suspension was treated with erucamide, oleamide, and camphor bromide each at a concentration of 1.5 mg·mL^−1^. The control group was treated with an equal volume of anhydrous ethanol. In the experiment, 4-week-old mulberry seedlings were inoculated with the treated bacterial suspension at a concentration of 1.2 × 10^8^ CFU·mL^−1^, respectively. Sterile water was used as a blank control, and each treatment was repeated three times [58]. Post-inoculation, the seedlings were transferred into a climate-controlled incubator (26 °C, 75% humidity, light intensity: 6500 Lux) for cultivation. By analyzing the degree of leaf wilting, the incidence of diseases can be monitored and recorded to evaluate the protective effect of root exudates on mulberry wilt disease.

### 4.9. Statistical Analysis

All experiments were systematically repeated a minimum of three times, consistently yielding similar results. Statistical analyses were carried out using the IBM Statistical Product and Service Solutions (SPSS) software, version 26.0. To assess whether there existed statistically significant differences among various treatment groups, a one-way analysis of variance (ANOVA) was performed using Systat software (Systat, Inc., Evanston, IL, USA), version 13.2. Furthermore, Dunnett’s post hoc test was utilized to compare mean differences at a significance level of *p* < 0.05, thereby establishing any significant variations among treatments in terms of gene expression.

## 5. Conclusions

According to the findings of our study, we have identified that three specific active substances in the root exudates exhibit a significant inhibitory effect on both the biofilm formation and motility of the *Rp*. Mulberry root exudates can induce a burst of reactive oxygen species (ROS), causing oxidative damage to the bacterial cells and affecting their growth activity. Treatment with camphor bromide increases the secretion of extracellular polysaccharides in the *Rp*, leading to increased cell membrane permeability. An analysis of the expression patterns of virulence-related genes showed that treatment with erucamide, oleamide, and camphor bromide significantly decreased the expression levels of *hrpB*, *hrpX*, *hrpF*, *ripAW*, *ripAE*, *Rs5-4819*, *Rs5-4374*, *ace*, *egl3*, and *pehB* in *Rp* strain MRS-5. Furthermore, under camphor bromide stress, the expression level of the global regulatory factor *phcA* gene was significantly decreased. Camphor bromide and oleamide affect the root growth of Mulberry seedlings, while erucamide weakens the pathogenicity of MRS-5 and has no significant effect on the root growth of mulberry seedlings. These substances can be used as plant-derived biocontrol agents for the prevention and control of mulberry wilt disease. This research provides insights into the regulation of the pathogenicity mechanisms of the mulberry bacterial wilt and offers an environmentally friendly and effective strategy for the development of control agents against mulberry bacterial wilt.

## Data Availability

The data presented in this study are available within the article and Supplementary Materials.

## Supplementary Materials

The following supporting information can be downloaded at: https://www.mdpi.com/article/10.3390/plants13040482/s1, Figure S1: Collection of root exudates from Mulberry seedlings; Figure S2: Total ion chromatogram of root exudates from Mulberry seedlings under different pH conditions; Table S1: Primer information of virulence-related genes of *Rp*.
