# Supplementary material for: Antibacterial Activity and Mechanism of Three Root Exudates from Mulberry Seedlings against Ralstonia pseudosolanacearum
Source: Plants (Basel). 2024 Feb 8;13(4):482. doi: 10.3390/plants13040482 (PMC10892386; doi:10.3390/plants13040482)
Supplement: Supplementary file 1 [file plants-13-00482-s001.zip › plants-2841169-supplementary.pdf]

Supplementary Materials

Antibacterial Activity and Mechanism of Three Root Exudates from Mulberry Seedlings against *Ralstonia pseudosolanacearum*

Ping Li [1,2], Siyi Wang [1], Mengyuan Liu [1], Xue Dai [1], Huicong Shi [1], Weihong Zhou [1,2], Sheng Sheng [1,2] and Fuan Wu [1,2,*]

[1] Jiangsu Key Laboratory of Sericultural Biology and Biotechnology, School of Biotechnology, Jiangsu University of Science and Technology, Zhenjiang 212100, China; lee_ping2020@163.com (P.L.); 221211802114@stu.just.edu.cn (S.W.); 211111802105@stu.just.edu.cn (M.L.); 231211801103@stu.just.edu.cn (X.D.); shihcc08@163.com (H.S.); zhouweihong2020@just.edu.cn (W.Z.); parasitoids@163.com (S.S.)

[2] Key Laboratory of Silkworm and Mulberry Genetic Improvement, Ministry of Agriculture and Rural Affairs, The Sericultural Research Institute, Chinese Academy of Agricultural Sciences, Zhenjiang 212100, China

* Correspondence: fuword@163.com

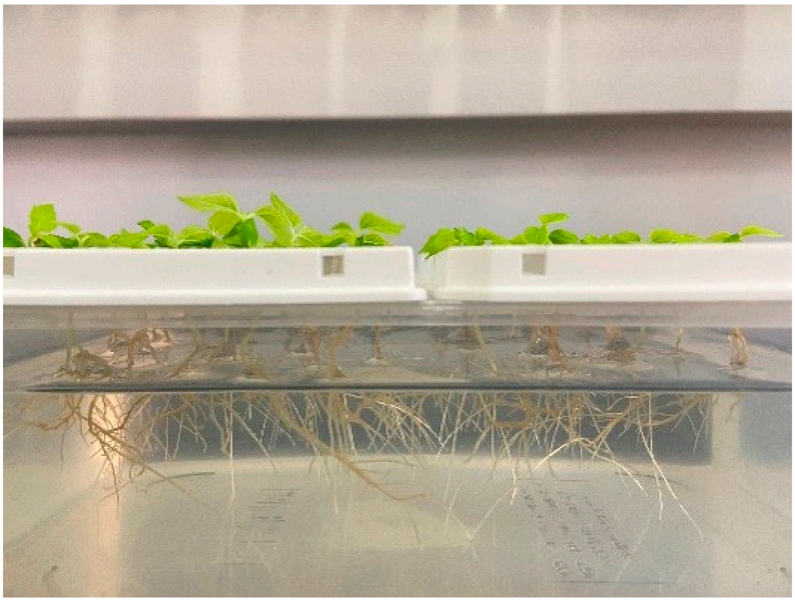

Figure S1. Collection of Root Exudates from “Feng Chi”. Mulberry Seedlings. This figure illustrates the growth status of mulberry seedlings following a 30-days.

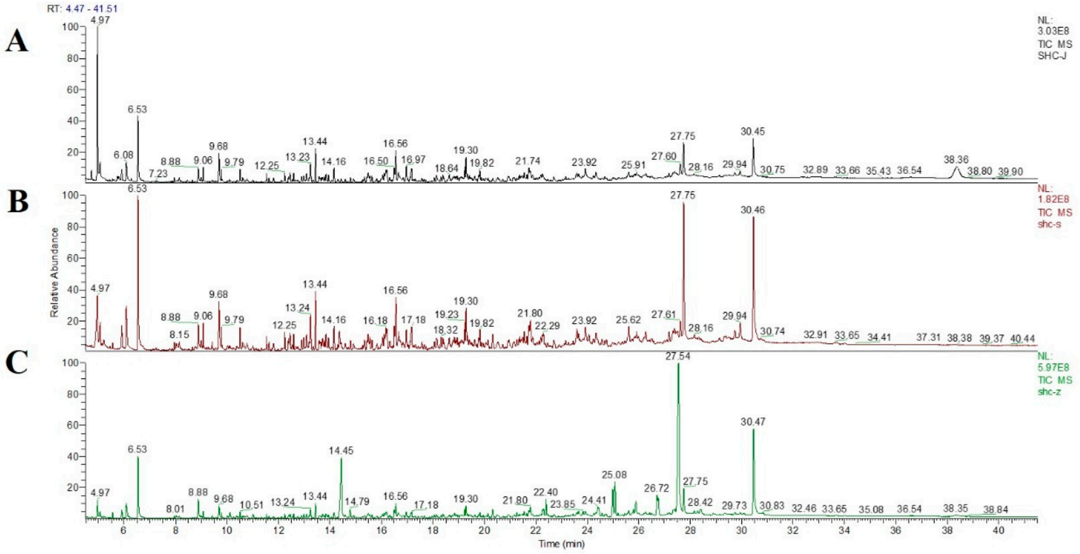

Figure S2. Total Ion Chromatogram of Root Exudates from Mulberry Seedlings under Different pH Conditions: (**A**) alkaline conditions; (**B**) Acidic conditions; (**C**) Neutrality Condition.

Table S1. Primer information of virulence-related genes of *R. pseudosolanacearum*.

Primer Name	Primer Sequence 5' to 3'	Product Size
16s-F	GACGACGAGTTCTACTGGGT	168 bp
16s-R	CACATACACCTCGACGAACG	
hrpB-F	GAGCATGCGTTCTGGCAATC	143 bp
hrpB-R	AGCAGGCTGAGGTATTCGG	
hrpX-F	GATTCACGCCCGACATCATC	125 bp
hrpX-R	GAATTCCATGGACGTCTCGC	
hrpF-F	CTGTTCTTCGCCATCGGTTC	117 bp
hrpF-R	CTTCTTGCGGTTGTACTCGG	
ripAW-F	CTGTCCGATATCCTGCCGAT	98 bp
ripAW-R	GCAATGTAGGCTCTTGCCTG	
ripAE-F	CGACCAGATGCACAAGAACG	135 bp
ripAE-R	TCGAGCGAATACCTGTCCG	
Rs5-1997-F	TGTGTTCAATCGGACCATGC	129 bp
Rs5-1997-R	TCGCAGGCATATCGCATTTC	
Rs5-4374-F	CGAGTACAACCGCAAGAAGG	126 bp
Rs5-4374-R	TCGGAATACCGGTACAGCTC	
Rs5-4819-F	GATTCACGCCCGACATCATC	183 bp
Rs5-4819-R	CAAGCGTACGAAGCTGCTC	
ace-F	CACCAGCAGAAGAAGATGCC	129 bp
ace-R	TTCCAGTTCCTTCGAGCCTT	
phcA-F	ATCCGGTCCTGAAGGATCAC	165 bp
phcA-R	CATGCACAGTCATGGTGGAG	
pehB-F	ATGTTCATGACCGACAACGG	179 bp
pehB-R	GACAACGTGAGGATGAACGG	
egl3-F	ACAAGAACAAGGGCATGAACC	
egl3-R	GATCACGTTGCCGTAGTAGC	188 bp
